# The Effects of Injection Site on the Reflux Following Intravitreal Injections

**DOI:** 10.4021/jocmr2009.12.1280

**Published:** 2009-12-28

**Authors:** Burak Turgut, Tamer Demir, Ulku Celiker

**Affiliations:** a Firat University School of Medicine, Department of Ophthalmology, Elazig, Turkey

## Abstract

**Background:**

This study aimed to investigate the effects of injection site on the reflux after intravitreal injection.

**Methods:**

One hundred and eighty eyes undergoing intravitreal injection including 0.1 ml of triamcinolone acetonide or bevacizumab or pegaptanib were divided to six groups (30 patients in each group) to compare the vitreal reflux after injection using superotemporal versus inferotemporal quadrant. The amount of intraoperative reflux was estimated by measuring the width of the subconjunctival bleb. An interventional, prospective, comparative clinical trial was applied.

**Results:**

The mean bleb width as the reflux amount after injection of three drugs was statistically less after the inferotemporal injection (1.50 ± 0.94 mm for triamcinolone acetonide, p < 0.001; 1.60 ± 1.07 mm for bevacizumab, p < 0.001; and 1.77 ± 0.94 mm for pegaptanib, p = 0.001) than those in eyes undergoing the superotemporal injection (3.20 ± 1.63 mm for triamcinolone acetonide; 3.07 ± 1.53 mm for bevacizumab; and 2.80 ± 1.32 mm for pegaptanib).

**Conclusions:**

The injection through inferotemporal quadrant provides statistically significant less vitreal reflux for intravitreal drug injection.

**Keywords:**

Intravitreal injection; Injection site; Reflux

## Introduction

The use of intravitreal (IVT) drug injections for the treatment of various refractory retinal diseases has increased rapidly in the current decade. Despite of the off-label use of IVT triamcinolone acetonide (TA) (Kenalog; Bristol-Myers Squibb, New York, NY) and bevacizumab injection, it has been reported that both drugs in intravitreal rate are efficient and safe agents in macular oedema and retinal neovascularization [1-6]. Previous clinical and experimental trial data have demonstrated that the therapeutic aptamer oligonucleotide pegaptanib sodium (Macugen; Eyetech Pharmaceuticals, New York, NY) and the monoclonal antibody fragment ranibizumab (Lucentis; Genentech, San Francisco, CA) are effective for the treatment of neovascular age related macular degeneration (AMD) [7-10].

Drug reflux is clinically important to drug efficacy or safety of IVT injections. The reflux has an important intraoperative complication in IVT injections because the problems that could be associated with a vitreous or drug reflux are the misplacement of substantial amount of the injected drug, the vitreous wick and the increase in the risk of endophthalmitis due to the entering of the bacteria from the ocular surface through injection site. It has been reported that the factors such as volume amount, injection speed, and length and direction of the scleral incision might have influence on amount of vitreal reflux after IVT injections. However, there was no report or consensus on the site or the quadrant location that used for injection [11-21]. Thus, in this study we aimed to investigate the effects of injection site on the reflux following IVT injections.

## Materials and Methods

### Subjects and design

One hundred and eighty eyes of 180 patients with various retinal disorders who were taken under follow-up in the retinal outpatient clinic of our university hospital were enrolled to this study. The study was designed as an interventional, prospective and comparative clinical trial. In this study, the indications for IVT treatment were proliferative diabetic retinopathy (n = 13), diabetic macular oedema (n = 53), macular oedema due to branch retinal vein occlusion (n = 24) and central retinal vein occlusion (n = 12), and neovascular age-related macular degeneration (n = 78).

All patients underwent a complete ophthalmologic and general examination. The tenets of the Helsinki declaration were followed throughout the study. Informed consent was obtained from each subject including detailed explanations of all procedures before participation in the study.

The patients with disorders such as connective tissue disease, degenerative myopia and scleritis, which refers that scleral thickness may be affected, the patients with obesity and blepharospasm of whom the disorders may affect the reflux, and the patients who underwent intravitreal injection or vitrectomy prior to the study were excluded from study.

Patients were divided to six groups: TA injection via the superotemporal quadrant (Group 1, n = 30), TA injection via the inferotemporal quadrant (Group 2, n = 30), bevacizumab injection via the superotemporal quadrant (Group 3, n = 30), bevacizumab injection via the inferotemporal quadrant (Group 4, n = 30), pegaptanib injection via the superotemporal quadrant (Group 5, n = 30), and pegaptanib injection via the inferotemporal quadrant (Group 6, n = 30).

### The pars plana injection technique

Before the injection is administered, Oxybuprocaine hydrochloride drop and 10% povidone-iodine wash using a flush injector were applied directly to the ocular surface, lid margins, and lashes. After a lid speculum was placed, an additional drop of povidone-iodine and topical anaesthetic was applied to the intended injection site. No instrumentation was performed for the globe fixation during the injection because the potential elevation of intraocular pressure due to fixation might influence reflux.

Injection was performed by the surgeon (B.T.) through the pars plana using a 27-gauge needle on a 1-ml tuberculin syringe in temporal quadrants 3 mm (pseudophakic eyes) to 4 mm (phakic eyes) from the limbus, and then 0.1 ml with 4 mg TA or 2.5 mg bevacizumab or 0.3 mg pegaptanib was injected into the mid vitreous cavity. The standard straight injection perpendicular to the sclera was slowly created after upward mobilization of the conjunctiva and the syringe needle was then gently withdrawn. In order to avoid vitreous wick syndrome, the conjunctiva shifted with a cotton-tipped applicator before injection and then, to minimize reflux, this applicator was softly applied over the scleral entry site to the needle withdrawn for about three seconds.

In all the six groups, the amount of vitreal reflux was assessed by measuring the width of the broadest conjunctival elevation of the subconjunctival bleb with a surgical calliper described by Rodriques [22]. Intraocular pressures (IOP) were measured 30 minutes later after the injection in all patients.

### Statistical analysis

Statistical analysis was performed using the Statistical Package for the Social Sciences version 13.0 (SPSS, Inc., Chicago, IL, USA). The Student t test was used to estimate differences within and among the study groups. Results were given as means + standard deviations. P value less than 0.05 was considered as statistically significant.

## Results

One hundred and thirty two patients were phakic, and 48 were pseudophakic. Sixty-one percent of patients were women and 39% were men. Mean age was 68.6 ± 7.2 years (range, 54 to 77 years) in Group 1, 65.4 ± 5.9 years (range, 56 to 76 years) in Group 2, 67.2 ± 8.3 years (range, 58 to 83 years) in Groups 3, 64.8 ± 9.2 years (range, 52 to 83 years) in Group 4 (range, 60 to 80 years), 66.6 ± 8.1 years (range, 51 to 74 years) in Group 5, and 69.6 ± 6.2 (range, 62 to 76 years) in Group 6.

The mean scales ± standard deviations (SD) of reflux width obtained with measuring the broadest conjunctival elevation after IVT injection were 3.20 ± 1.63 mm in Group 1, 1.50 ± 0.94 mm in Group 2, 3.07 ± 1.53 mm in Group 3, 1.60 ± 1.07mm in Group 4, 2.80 ± 1.32 mm in Group 5, and 1.77 ± 0.94 mm in Group 6 (Table 1).

**Table 1:** The Reflux Amounts and the IOP Measurements After the Injection (Mean ± SD) in the Study Groups

Groups	Subconjunctival Bleb Diameters(Mean ± SD)	IOP Measurements
		(Mean ± SD)
Group 1 (IVTA-Superotemporal Injection)	3.20 ± 1.63 mm	17.6 ± 4.1 mm Hg
Group 2 (IVTA-Inferotemporal Injection)	1.50 ± 0.94 mm	19.0 ± 4.7 mm Hg
Group 3 (IVB-Superotemporal Injection)	3.07 ± 1.53 mm	15.3 ± 3.1 mm Hg
Group 4 (IVB-Inferotemporal Injection)	1.60 ± 1.07 mm	16.8 ± 3.5 mm Hg
Group 5 (IVP-Superotemporal Injection)	2.80 ± 1.32 mm	18.4 ± 4.4 mm Hg
Group 6 (IVP-Inferotemporal Injection)	1.77 ± 0.94 mm	20.1 ± 5.2 mm Hg
P value	P < 0.05 Group 1 vs. Group 2	P > 0.05 for all the groups
	P < 0.05 Group 3 vs. Group 4	
	P < 0.05 Group 5 vs. Group 6	

IVTA: Intravitreal Triamcinolone; IVB: Intravitreal bevacizumab; IVP: Intravitreal pegaptanib.

The mean vitreal reflux width of the broadest conjunctival elevation after TA injection was statistically greater with injection in superotemporal quadrant in Group 1 (3.20 ± 1.63 mm) compared with the injection in inferotemporal quadrant in Group 2 (1.50 ± 0.94. mm) (p < 0.01).

The mean vitreal reflux width of the broadest conjunctival elevation after bevacizumab injection was statistically greater with injection in superotemporal quadrant in Group 3 (3.07 ± 1.53 mm) compared with the injection in inferotemporal quadrant in Group 4 (1.60 ± 1.07 mm) ( p < 0.001).

The mean vitreal reflux width of the broadest conjunctival elevation after bevacizumab injection was statistically greater with injection in superotemporal quadrant in Group 5 ( 2.80 ± 1.32 mm) compared with the injection in inferotemporal quadrant in Group 6 ( 1.77 ± 0.94 mm) ( p = 0.001).

**Figure 1:**
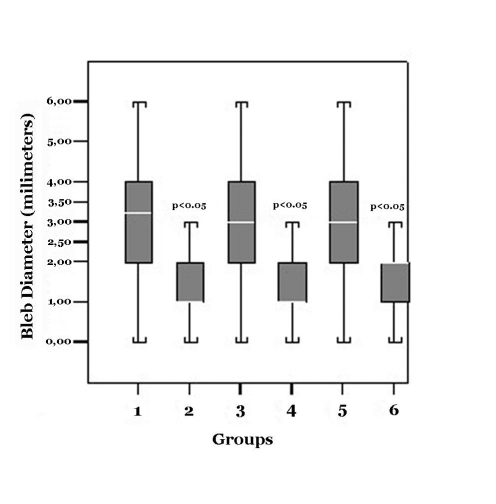
Box plot Graphics demonstrates the amount of reflux after the injection

When all groups were compared, it has been found that the mean vitreal reflux width with injection in superotemporal quadrants (Group 1, 3 and 5) was significantly higher compared to those in inferotemporal quadrants (Group 2, 4 and 6) (p < 0.001) (Fig. 1). Neither the differences in reflux among Groups 1, 3 and 5 nor among Groups 2, 4 and 6 were found to be statistically significant (p > 0.05).

Along the follow-up period lasting the mean 24 weeks, no complication due to IVT injection was detected in the patients. The IOP difference among all the groups was not significant (p > 0.05). The mean IOP measurements and Standard deviations were given in Table 1.

Subconjunctival hemorrhage following injection was occurred in 3 patients in group 1, in 2 patients in group 2, in one patient in group 3, in 2 patients in group 4, in 2 patients in group 5, and in 3 patients in group 6. However, this did not influence the amount of reflux and the assessment of width of bleb because these patients had no reflux.

There was either no significant difference among the mean age and the numbers of phakic and pseudophakic patients in the groups.

## Discussion

The use of intravitreal injections has been now commonly chosen by retina specialists in the treatment of retinal disease, and it provides numerousadvantages over other methods of administering drugs to the posterior segment such as efficient intraocular concentrations and low systemic exposure. Despite that the descriptions of IVT injection techniques were insufficient, the variables were described in the injection procedure such as the prophylactic and postoperative antibiotic therapy, the anaesthesia and antisepsis of the injection site, the controlling postoperative elevations in IOP, the injection quadrant, distance from limbus, and the provision of eye immobilization [12-16]. It has been also reported that the factors such as volume amount, injection speed, length and direction of the scleral incision, the needle size, the consequential rise in IOP, and the injection technique used might have influence on amount of vitreal reflux after IVT injections. However, there was no report or consensus on the site or the quadrant location that used for injection except the direction of the needle towards the centre of vitreous cavity [11-21].

During removal of the needle following IVT injection, vitreous, liquefied vitreous or fluid can reflow through the needle incision into the subconjunctival space. The reflux after intravitreal injection can be observed in about 20% of injections [17]. The problems that could be associated with a vitreous or drug reflux are the misplacement of substantial amount of the injected drug, the vitreous wick syndrome and the increase in the risk of endophthalmitis due to the entering of the bacteria from the ocular surface through injection site [18,19].

Although a straight needle path, pointing at the centre of the eyeball is usually recommended, in order to lower the risk of vitreous reflux, it has been recommended of using sharp smallgauge needles (27 gauge needle for Triamcinolone and 30 gauge needle for other drugs) or using a slightly angled scleral path with the injection needle, a short scleral tunnel for injection and pulling the conjunctiva over the injection site [18-21]. Recently, Rodriques et al [22] reported that the tunnelled scleral incision provides less vitreal reflux following IVT drug injection. Additionally, Lopez-Guajardo et al [23] demonstrated that the use of the oblique injection technique could reduce drug loss after intravitreal pegaptanib injection. In case that we focused on the effect of injection quadrant on the reflux, the straight scleral injection was created in all the patients.

It was also considered that the vitreous changes due to aging may influence the reflux. However, there was no significant difference among the mean age in the study groups.

Concerning with the location of the needle penetration into the vitreous, most studies reported that penetration was created in 3 mm in aphakic or pseudophakic eyes and in 4 mm in phakic eyes posterior to the corneoscleral limbus. Location of the injected material may strongly affect distribution of the compound within the vitreous, bioavailability and retinal toxicity [1, 2, 7, 16-18]. In our study, all the incisions were made between 3.0 and 4.0 mm posterior to the limbus, depending on the lens status.

In IVT injections, the volume of administered compound was generally suggested 0.1 ml [24]. In this study, the standardized volume of administered compound for all injections was 0.1 ml.

The use of 27 or 30-gauge needles measuring between 12.7 and 15.75 mm long and the advancement of the needle 6 - 7 mm toward the centre of the eyeball to avoid injection along the anterior vitreous surface are usually recommended [18]. In order to standardize, 27-gauge needles with 12.7 mm length were used in incision and the needles were advanced 6 mm towards mid vitreous cavity.

In our study it was found that vitreal reflux was lesser in injection using inferotemporal quadrant compared to those in superotemporal quadrant. To the best knowledge, there is no data involving the thickness difference among different scleral quadrants. Only, it has been known that the human sclera gradually thickens from the equatorial regions towards the posterior to reach a maximum thickness [25].

On the other hand, a variety of diseases or conditions including vasculitis, ocular surface diseases, ocular infections, scleritis, episcleritis, collagen or connective tissue disorders, glaucoma, degenerative myopia, prior trabeculectomy with MMC or vitrectomy, may cause the thinning of scleral wall or the decreasing in scleral rigidity [26-29]. Thus, the patients with disorders mentioned above which may affect the scleral thickness and rigidity were excluded from study.

The results from our study may be due to that thickness of the sclera in inferotemporal region is more than those in superotemporal. If this is demonstrated, less reflux in this quadrant may be explained. However, the measuring of the scleral thickness in different quadrants using preferably ultrasound biomicroscopy may explain our results.

In conclusion, we consider the usage of the inferotemporal quadrant for the performing intravitreal injections because the use of this site can result in reduced drug loss after intravitreal injections. When the amount of the drug within reflux should be detected, the effect of reflux on the efficacy of the drug administered through intravitreal rate could be understood. Future studies in humans and animals are needed to determine the relationship between the vitreal reflux bleb and amount of drug volume loss after IVT injection. The next step is to investigate the reflux degree in pseudophakic and phakic eyes, the possible effect of the usage of 30-gauge needle for injection, of the laterality of the eye injected and gender differences on the degree of reflux in more comprehensive studies.
